# The Differential Paracrine Role of the Endothelium in Prostate Cancer Cells

**DOI:** 10.3390/cancers14194750

**Published:** 2022-09-29

**Authors:** Verónica Torres-Estay, Michalis Mastri, Spencer Rosario, Patricia Fuenzalida, Carolina E. Echeverría, Emilia Flores, Anica Watts, Javier Cerda-Infante, Viviana P. Montecinos, Paula C. Sotomayor, Julio Amigo, Carlos Escudero, Francisco Nualart, John M. L. Ebos, Dominic J. Smiraglia, Alejandro S. Godoy

**Affiliations:** 1 Department of Chemical and Biological Sciences, Universidad Bernardo O’Higgins, Santiago 8370993, Chile; 2 Department of Cancer Genetics and Genomics, Roswell Park Comprehensive Cancer Center, Buffalo, NY 14263, USA; 3 Department of Physiology, Pontificia Universidad Católica de Chile, Santiago 8331150, Chile; 4 Centro de Biología Celular y Biomedicina (CEBICEM), Facultad de Medicina y Ciencia, Universidad San Sebastián, Santiago 7510156, Chile; 5 Centro de Investigación e Innovación Biomédica, Universidad de los Andes, Santiago 7620001, Chile; 6 Department of Urology, Roswell Park Comprehensive Cancer Center, Buffalo, NY 14263, USA; 7 Department of Hematology Oncology, Pontificia Universidad Católica de Chile, Santiago 8331150, Chile; 8 Department of Urology, Pontificia Universidad Católica de Chile, Santiago 8331150, Chile; 9 Department of Basic Science, Faculty of Sciences, Universidad del Bio-Bio, Chillan 3800708, Chile; 10 Group of Research and Innovation in Vascular Health (GRIVAS Health), Chillan 3800708, Chile; 11 Departamento de Biología Celular, Facultad de Ciencias Biológicas, Universidad de Concepción, Concepción 4070386, Chile; 12 Department of Medicine, Roswell Park Comprehensive Cancer Center, Buffalo, NY 14263, USA

**Keywords:** angiocrine effect, endothelial cells, cancer, prostate cancer, microenvironment

## Abstract

**Simple Summary:**

A growing body of literature supports the concept that a tumor mass is under the strict control of the microvascular endothelium and that the perfusion of oxygen and nutrients by capillary vessels to the tumor mass is reinforced by potent paracrine activity from the vascular endothelial cells. In our study, we investigate the biological and molecular implications of the paracrine crosstalk between vascular endothelial cells and prostate cancer cells. Our results indicate that the endothelial cells were able to secrete molecular signals that promote the proliferation and growth of low and highly aggressive prostate cancer cells and selectively increased the migration, invasion and metastatic potential of highly aggressive prostate cancer cells. The molecular analyses indicated that endothelial cells induced a differential effect on gene expression profile when comparing low versus highly aggressive prostate cancer cells, causing an enrichment of epigenetic changes in migratory pathways in highly aggressive prostate cancer cells. In conclusion, our results indicate that endothelial cells release signals that favor tumor growth and aggressiveness and that this interaction may play an important role in the progression of prostate cancer.

**Abstract:**

The survival of patients with solid tumors, such as prostate cancer (PCa), has been limited and fleeting with anti-angiogenic therapies. It was previously thought that the mechanism by which the vasculature regulates tumor growth was driven by a passive movement of oxygen and nutrients to the tumor tissue. However, previous evidence suggests that endothelial cells have an alternative role in changing the behavior of tumor cells and contributing to cancer progression. Determining the impact of molecular signals/growth factors released by endothelial cells (ECs) on established PCa cell lines in vitro and in vivo could help to explain the mechanism by which ECs regulate tumor growth. Using cell-conditioned media collected from HUVEC (HUVEC-CM), our data show the stimulated proliferation of all the PCa cell lines tested. However, in more aggressive PCa cell lines, HUVEC-CM selectively promoted migration and invasion in vitro and in vivo. Using a PCa-cell-line-derived xenograft model co-injected with HUVEC or preincubated with HUVEC-CM, our results are consistent with the in vitro data, showing enhanced tumor growth, increased tumor microvasculature and promoted metastasis. Gene set enrichment analyses from RNA-Seq gene expression profiles showed that HUVEC-CM induced a differential effect on gene expression when comparing low versus highly aggressive PCa cell lines, demonstrating epigenetic and migratory pathway enrichments in highly aggressive PCa cells. In summary, paracrine stimulation by HUVEC increased PCa cell proliferation and tumor growth and selectively promoted migration and metastatic potential in more aggressive PCa cell lines.

## 1. Introduction

Prostate cancer is the second leading cause of cancer related death in men worldwide [1]. It is also the most common non-cutaneous cancer in that population. While the standard of care for PCa is androgen deprivation therapy, it does not offer permanent remission from androgen-sensitive PCa, which develops into a more aggressive castration-resistant CR-PCa disease [2].

Angiogenesis is involved in a solid tumors ability to grow and spread. In prostate cancer, the extent of angiogenesis is commonly expressed as microvessel density (MVD). MVD relates clinically with Gleason score and pathological stage [3,4]. Previous research has suggested MVD is an independent factor as a predictor of recurrence and therefore patient survival [5,6,7]. Clinical trials [8,9] in PCa using anti-angiogenic agents as monotherapies or in combination with other chemotherapeutic agents have been inconclusive or produced disappointing results. A conceivable explanation for the lack of results with this treatment modality is that the remaining ECs are able to directly regulate tumor growth and progression by releasing growth factors [10,11,12,13,14,15,16,17,18]. This is supported by in vitro research [19,20,21], where co-cultured EC/cancer cells demonstrated increased the proliferation, invasion, migration and survival of the cancer cells. It was also observed in PCa cell lines [21,22,23], which have been documented to respond to the paracrine effect of endothelial cells. In these studies, however, it is hard to distinguish between the contribution of the endothelial paracrine effect and the effect attributable to cell–cell interaction. It would be beneficial to gain a better understanding of the extent and mechanism by which ECs modulate PCa. In this work, we examine the impact of contact-independent paracrine signaling effects of HUVEC cells on various PCa tumor cell lines that varied in aggressiveness and in implanted xenograft tumor models. Our results show that conditioned media (CM) from HUVEC cells induces a myriad of transcriptomic and proteomic changes in PCa cells related to pro-migratory and pro-survival pathways. We found that CM from HUVEC cells could stimulate tumor migration and invasion, both in vitro and in vivo, with greater effects observed in more aggressive PCa cell variants. Together, these results suggest that aggressive PCa may be intrinsically ‘primed’ for the EC-induced stimulation of local and metastatic spreading, and potentially provide a reason for the diminished efficacy of antiangiogenic treatments in the treatment of patients with PCa.

## 2. Materials and Methods

### 2.1. Cell Cultures

Primary cultures of HUVEC cells were prepared, according to previously published protocols [24], from fresh umbilical cords from normal pregnancies. Signed informed consent was requested from all participants. The human prostate cell lines RWPE-1, LNCaP and PC-3 were commercially obtained from the American Type Culture Collection (ATCC). The cancer cell line LNCaP-C4-2 was kindly donated by Dr. Sergio Oñate, University of Concepción, Concepción, Chile. See the Supplemental Experimental Procedures for details.

### 2.2. Conditioned Media

Endothelial cells from human origin, HUVEC, HMEC-1 and HCMEC-D3, were grown to 90% confluence, and then washed three times with phosphate-buffered solution (PBS) and cultured for 36 h in growth medium supplemented with 1% fetal bovine serum (FBS). The conditioned media (CM) was collected and processed according to previously published protocols [11,25,26]. After preparation, CM was stored in aliquots at −80 °C.

### 2.3. Animals Models

For in vivo studies (NSG mice and zebrafish embryos), three different experimental settings were tested: (1) PC3 cells injected alone as a control condition; (2) PC3 cells co-injected with HUVEC; or (3) PC3 cells pre-incubated for 48 h with CM from HUVEC cells prior to injection. All animal experiments were conducted according to the guidelines and regulations of the scientific ethical committee for the care of animals and environment of Pontifical Catholic University of Chile (Santiago, Chile). See Supplementary Material and Methods S1 for details.

### 2.4. Cell Proliferation Assays

Cell proliferation was evaluated using CellTiter 96 AQueous Non-Radioactive Cell Proliferation Assay (Promega, Madison, WI, USA) and immunofluorescence staining of the proliferation marker Ki-67. See Supplementary Material and Methods S1 for details.

### 2.5. Transwell Migration and Invasion Assays

Migration and invasion assays were performed using the CytoSelect 96-Well Cell Migration and Invasion Assay (Cell Biolabs, Inc., San Diego, CA, USA). Experiments were performed according to the manufacturer’s instructions. See Supplementary Material and Methods S1 for details.

### 2.6. Zebrafish Xenograft Model

Two days post-fertilization (dpf), zebrafish embryos were injected into the middle of the embryonic yolk sac region with approximately 250 PCa cells (PC-3 and LNCaP). Embryos were imaged individually at 3 days post-injection using an inverted wide-field fluorescence microscope (DM IL LED, LEICA, Wetzlar, Germany). Cell fluorescence (red pixels) was measured in the tail of the fishes and quantified using ImageJ software. See supplementary information for details.

### 2.7. Cell Line-Derived Xenograft Model

NSG mice were injected subcutaneously with 1 × 10^6^ PC3 cells for control conditions, 1 × 10^6^ PC3 cells preincubated with CM from HUVEC cells for 48 h, or a mixture of 1 × 10^6^ PC3 cells and 2 × 10^5^ HUVECs cells (5:1). Tumor growth was monitored by measuring the length and width of tumor mass at the inoculation site with a caliper. See Supplementary Material and Methods S1 for details.

### 2.8. Immunohistochemistry

Immunostaining analyses of Ki-67, CD31, cleaved caspase-3 and VEGF were performed as previously described [27,28]. See Supplementary Material and Methods S1 for details.

### 2.9. Human Cytokine Array

Cytokines and chemokines present in the CM from HUVEC and in the cell culture media from LNCaP and PC3 cell lines previously exposed to conditioned medium from HUVEC cells for 48 h, were determined using the Proteome Profiler Human XL Cytokine Array Kit (R&D systems, Minneapolis, MN, USA) in accordance to the manufacturer’s instruction (Supplementary Table S1). Cell culture media from LNCaP and PC3 was collected 48 h after the removal of CM from HUVEC. Average signal (pixel density) of three independent CM(s) from HUVEC and three independent cell culture media were determined using ImageJ software. Expression level changes higher than a 1.5-fold were considered significant. Protein–protein interaction of secreted proteins from CM from HUVEC cells was generated using the STRING (Search Tool for the Retrieval of Interacting Genes/Proteins) database [29].

### 2.10. Expression Analysis

Expression profiling was carried out by the Genomic shared resource at Roswell Park Comprehensive Cancer Center, Buffalo NY. LNCaP and PC3 cell lines were treated with a mix of CM obtained from three independent HUVEC cultures. Genes with adjusted *p*-values of less than 0.05 and greater than a 1.5-fold change/less than a 1.5-fold change in expression were considered differentially expressed. DEGs were then used to construct a pre-ranked list for Gene Set Enrichment Analysis (GSEA) [30]. Ranks were assigned by multiplying the −log (adjusted *p* value) and the fold change, together. GSEA pre-ranked was then performed on these lists of enriched pathways. The results of the GSEA analysis were used to generate networks in Cytoscape [31], with the implementation of EnrichmentMap and AutoAnnotate plug-ins. See Supplementary Material and Methods S1 for details.

### 2.11. Statistical Analysis

Data were assayed by unpaired Student’s *t*-test and ANOVA test using GraphPad Prism statistical analysis software. A level of * *p* < 0.05 or ** *p* < 0.01 was regarded as statistically significant.

## 3. Results

### 3.1. HUVEC-CM Increases Proliferation, Migration and Invasion in Aggressive PCa Cell Lines

**Figure 1:**
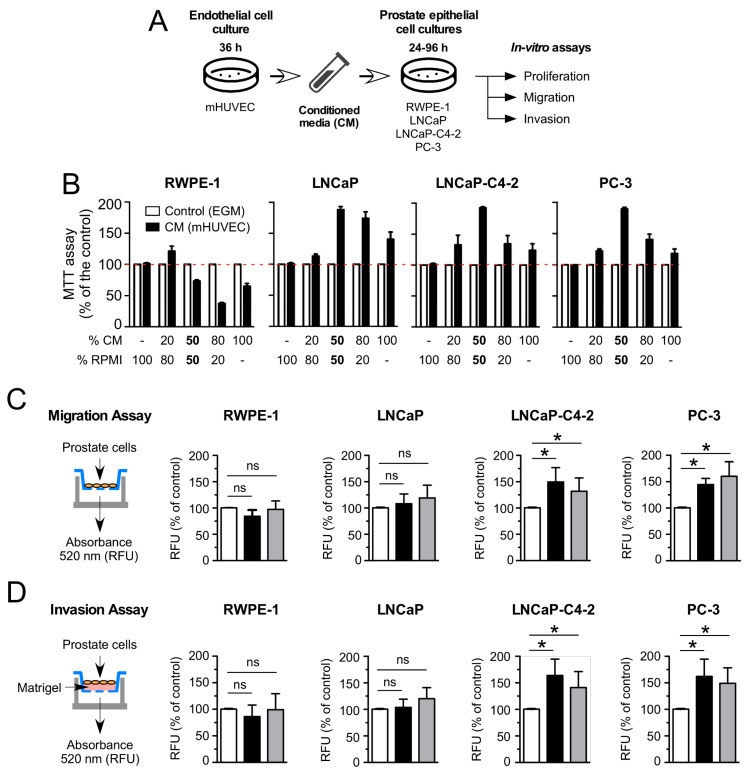
HUVEC-CM differentially increases the migration and invasion of aggressive PCa cell lines in vitro. (**A**) Schematic representation of the experimental procedures to obtain conditioned media (CM) from HUVEC and the in vitro assays. (**B**) Analysis of the effect of increasing concentration of CM (0, 10, 20, 50, 80 and 100%) isolated from primary cultures of HUVEC after mixture with standard PCa cell media supplemented with 5% FBS, on the cell proliferation of RWPE-1, LNCaP, PC3 and LNCaP-C4-2 cells using the MTT assay. Control condition for all experimental approaches was fresh endothelial culture medium (without FBS or growth factors) added to the standard RMPI medium in a 50/50 proportion (* *p* ≤ 0.05; *n* = 3). (**C**,**D**) Analysis of the effect of CM on migration and invasion. The following experimental conditions were used: RWPE-1, LNCaP, PC3 and LNCaP-C4-2 cells were seeded in the upper chamber (without fetal bovine serum (FBS)) in the presence of RPMI medium (control, white bars), conditioned media (CM, black bars), and previously incubated with CM for 48 h before seeding (Pre-CM, grey bars). The 10% FBS was added to the lower chambers as a chemo-attractant substance. (**C**) After 20 h, the migratory cells that passed through the polycarbonate membrane were lysed and quantified using fluorescent dye (* *p* ≤ 0.05; *n* = 3). (**D**) After 48 h, invasive cells that passed through the layer of Matrigel and the polycarbonate membrane were lysed and quantified using fluorescent dye. (*n* = 3; * *p* ≤ 0.05 *t*-test). All experiments were performed in triplicate. Values in mean ± SD.

The effect of HUVEC-CM was evaluated in benign prostate epithelial (RWPE-1) and in PCa (LNCaP, LNCa-C4-2 and PC-3) cell lines that represent phenotypes of different degrees of aggressiveness (LNCaP < LNCa-C4-2 < PC-3) (Figure 1A). Proliferation was examined using MTT (Figure 1B) and Ki-67 immunostaining (Supplementary Figure S1) analyses. Primary cultures of HUVEC were isolated and utilized as a model of endothelial cells due to their specific characteristic to express functional androgen receptor (AR) [24] comparably to human prostate endothelial cells (HPECs) [32]. Cell viability of all tested PCa cell lines was uniformly and significantly stimulated by HUVEC-CM (Figure 1B). Interestingly, the viability of the benign RWPE-1 cell line was not stimulated by HUVEC-CM (Figure 1B). The effect of HUVEC-CM on PCa cell migration and invasion were analyzed using transwell (Figure 1C,D) and wound healing assays (Supplementary Figure S2). HUVEC-CM potentiated the migration (Figure 1C and Supplementary Figure S2) and invasion (Figure 1D) of the highly aggressive LNCa-C4-2 and PC-3 PCa cell lines; however, HUVEC-CM did not affect these capacities of the less aggressive LNCaP PCa cell line or the benign RWPE-1 cell line (Figure 1C,D). Interestingly, LNCa-C4-2 or PC-3 cell lines treated with HUVEC-CM retained their increased migratory and invasive phenotypes even after seeded without HUVEC-CM (Figure 1C,D; grey bars). Together, our results indicated that HUVEC-CM seems to selectively promote cell migration and invasion of the more aggressive PCa cell lines, which may reflect the increased ability of these cells to respond to a broader range of signals/factors present in the HUVEC-CM. As a proof of concept for the universality of the effect of endothelial cells on PCa cells, we analyzed the effect of conditioned media isolated from the human microvascular endothelial cell line, HMEC-1 (HMEC-1-CM) and the human blood–brain barrier microvascular endothelial cells, HCMEC-D3 (HCMEC-D3-CM), on the proliferation and migration of LNCaP and PC-3 PCa cell lines (Supplementary Figure S3). Our results indicate that HMEC-1-CM did not affect proliferation, but significantly increased the migration of both PCa cell lines (Supplementary Figure S3). On the contrary, HCMEC-D3-CM did not affect proliferation or migration in both PCa cell lines (Supplementary Figure S3), which indicates a moderate degree of universality of the angiocrine effect of human endothelial cells on PCa cells.

### 3.2. HUVEC-Mediated Paracrine/Angiocrine Effects Enhance Migration and Growth of Tumors In Vivo

**Figure 2:**
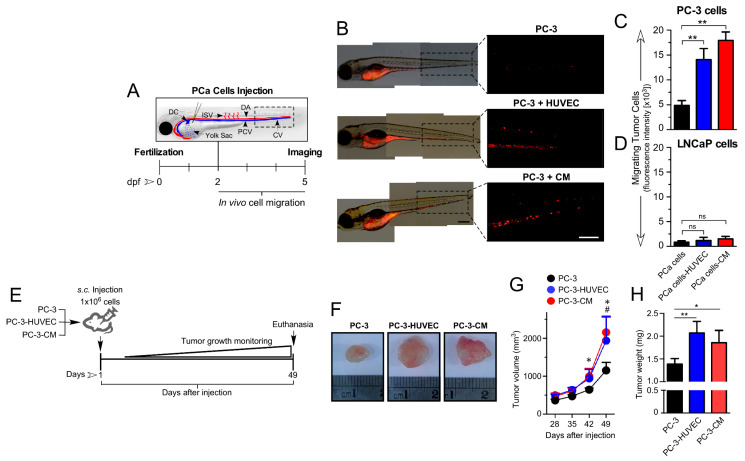
HUVEC-CM differentially promotes cell migration and tumor growth of aggressive PCa cell lines in vivo. (**A**) Schematic representation of the experimental conditions for the injection of PCa cells into the zebrafish model. PCa cells, marked with red cell tracker, were injected alone, co-injected with HUVEC cells (5:1 PCa:HUVEC cells) or preincubated for 48 h with CM from HUVEC before injection, into the yolk of zebrafish embryos at 48 h post fertilization (hpf). (**B**) Representative images of zebrafish at 72 h post-injection with red cell tracker-labeled PC-3 cells. Quantitation of the intensity of the red cell tracker fluorescence associated with migratory PC-3 (**C**) or LNCaP (**D**) cells in the CV (Caudal vein) of zebrafishes was determined by ImageJ software. (*n* = 3; * *p* < 0.05, ** *p* ≤ 0.01 *t*-test). (**E**) Schematic representation of the experimental conditions for the subcutaneous injections of PC-3 cells into NSG mice. PC-3 cells were injected subcutaneously alone (1 × 10^6^), co-injected with HUVEC cells (5:1 PCa:HUVEC cells) or pre-incubated for 48 h with CM from HUVEC cells before injection, in a mixture with Matrigel (1:1) for a total injection volume of 100 μL. (**F**) Representative images of surgically resected subcutaneous tumors for the three experimental conditions. (**G**) Average tumor volume from each experimental condition was measured weekly through the duration of the experiment. Tumor volume was determined by measuring the major (L) and minor (W) diameters with an electronic caliper, and volume calculated according to the formula: tumor volume= L × W × W/2. (*n* = 11; * *p* ≤ 0.05 versus PC3-CM, # *p* ≤ 0.05 versus PC3-HUVEC ANOVA test). (**H**) Average tumor weight (mg) was measured after surgical resection of tumors. (*n* = 11; * *p* ≤ 0.05, *t*-test). Values in mean ± SD.

Next, we evaluated the in vivo effects of HUVEC on PCa cell lines using the zebrafish embryo and the immunodeficient NSG mouse models (Figure 2A–E). Thus, in both models of xenografts, we injected PCa cells (used as the control). In order to analyze the effect of endothelial cells, we co-engrafted endothelial cells with cancer cells or pre-treated cancer cells with HUVEC-CM for 48 h before the injection. In the zebrafish embryo model, the androgen-insensitive PC-3 cells pre-treated with HUVEC-CM or co-engrafted with HUVEC cells exhibited a significant increase in their migratory capacity toward the trunk and tail of the embryos compared to the control conditions (Figure 2B,C). Confirming what was observed in the in vitro assays, the androgen-sensitive LNCaP cells did not migrate from the injection site of the zebrafish (Figure 2D). The transplantation of PCa cells into NSG mice pre-treated with HUVEC-CM or co-injected with HUVEC cells formed tumors that were larger in size (Figure 2F), volume (Figure 2G) and weight (Figure 2H) when compared to the control conditions (PC3 alone). No significant differences were observed for tumors formed by PC-3 cells pre-treated with HUVEC-CM and co-injected with HUVEC cells, suggesting that most of the effect of HUVEC cells on PC-3 tumor growth could be explained by an angiocrine/paracrine mechanism, with little to no additional effect mediated by cell–cell contact between HUVEC and PC-3 cells.

### 3.3. HUVEC-CM Increases PCa Cell Proliferation, Microvascular Density and VEGF-A Expression in PC-3 Cell-Line-Derived Xenograft Tumors

**Figure 3:**
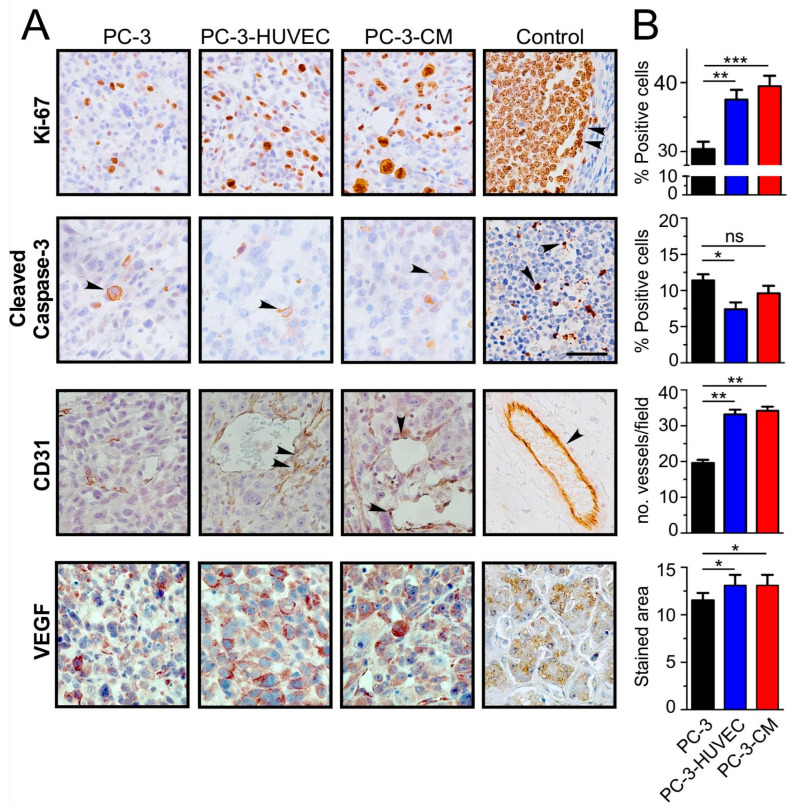
HUVEC-CM increases PCa proliferation and microvessel density in vivo. PC3 cells were injected subcutaneously alone (1 × 10^6^), co-injected with HUVEC (5:1 PCa:HUVEC cells), or pre-incubated for 48 h with CM from HUVEC before injection in a mixture with Matrigel (1:1) for a total injection volume of 100 μL. (**A**) Representative images of the immunohistochemical analysis of Ki-67, cleaved caspase-3, CD31 and VEGF expression in tissue sections of PC-3 cell line-derived xenograft tumors. (**B**) Quantitation of the number of positive cells per field for Ki-67 and cleaved caspase-3, number of vessels per field (CD31) and immunostained area (VEGF) in tissue sections of PC-3 cell-line-derived xenograft tumors was determined using ImageJ software. Positive controls for the expression of Ki-67 and cleaved caspase-3 were human tonsil, CD31 and VEGF, mouse adipose tissue, and human liver, respectively (*n* = 8; * *p* ≤ 0.05, ** *p* ≤ 0.01, *** *p* ≤ 0.001 *t*-test) Values in mean ± SD.

The biological mechanisms underlying the paracrine effect of HUVEC on PC-3 tumor growth in vivo were assessed using immunohistochemical analyses (Figure 3A,B). The number of tumor cells that expressed the proliferation marker Ki-67 was increased significantly in both PC-3 cells pre-treated with HUVEC-CM and in PC-3 cells co-injected with HUVEC cells. Conversely, while the level of apoptosis in the tumor tissue specimens generally was low, a significant decrease in cleaved caspase-3 immunostaining was observed only in PC3 cells co-injected with HUVEC.

A major focus of this study was to determine whether endothelial cells can modulate the pro-angiogenic capacity of PC-3 tumor cells in vivo (Figure 3A,B). Both, MVD (Figure 3B, nro. vessels/field) and VEGF-A (Figure 3B, stained area) expressions were significantly increased in tumors produced by co-injection of PC-3 cells with HUVEC or PC-3 cells pre-treated with HUVEC-CM. Since there were no significant differences between these two experimental conditions, we hypothesize that HUVEC cells increase tumor growth predominantly through a paracrine communication that enhances both the proliferation and pro-angiogenic potential of PCa cells.

### 3.4. Human Endothelial Cells Enhance Metastasis of PC-3 Cell-Line-Derived Xenograft Tumors

**Figure 4:**
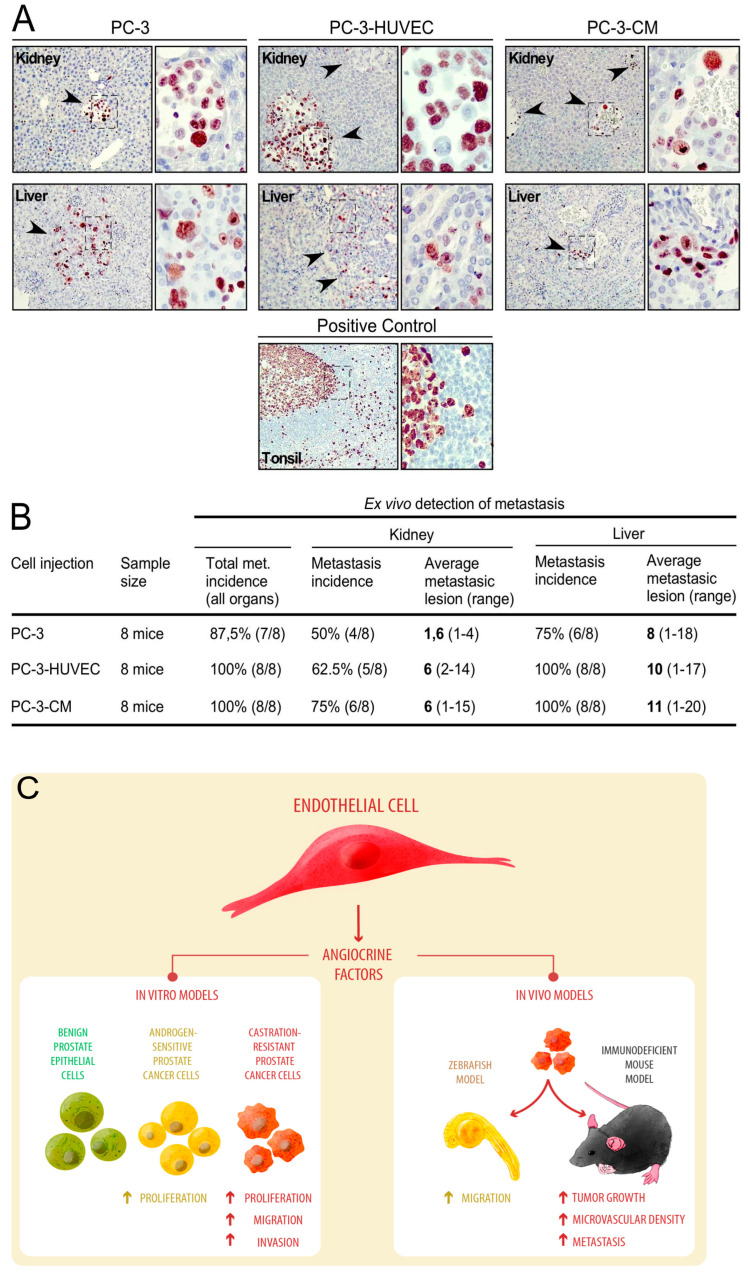
HUVEC-CM increased metastatic foci in host tissues of mice xeno-transplanted with PC-3 cells. (**A**) Representative images of metastatic foci in host kidney and liver tissues. Metastatic PC-3 cells were immunohistochemically detected using an anti-human Ki-67 antibody. Human tonsil was used as positive control for human Ki-67 expression. The tonsil has a characteristic pattern for this marker, since the surface of the epithelium includes a high positive (parabasal layer), low positive (intermediate layer) and negative (basal and superficial layers) zones. (**B**) Quantification of the number of mice with metastasis (metastasis incidence) and the number of metastatic foci per field in each organ (“Average metastatic lesion (range)” (*n* = 8). (**C**) Proposed model of the paracrine effects of endothelial cell CM on prostate epithelial cell lines in in vivo and in vitro experiments.

The detection of metastatic foci in mouse tissues was achieved using the immunostaining analyses of Ki-67 of human origin (Figure 4A). The incidence of total metastasis (sum of all organs) was slightly higher from tumors of PC-3 cells pre-treated with HUVEC-CM or PC-3 cells co-injected with HUVEC when compared to tumors of PC-3 alone (Figure 4B). Furthermore, when the incidence of metastasis was analyzed in individual organs (kidney and liver), the number of metastatic foci (Figure 4B) was consistently higher in both PC-3 cells pre-treated with HUVEC-CM or PC-3 cells co-injected with HUVEC compared to PC-3 alone (Figure 4B). Our data support the concept that HUVEC cells can promote the migration, invasion and metastatic potential of PCa cells through a paracrine mechanism, probably mediated by multiple signals/factors released by the endothelium, which impact human PCa cells differentially (Figure 4C).

### 3.5. Proteomic Analysis of The Primary Signals/Factors Involved in Intercellular Communication from HUVEC-CM

**Figure 5:**
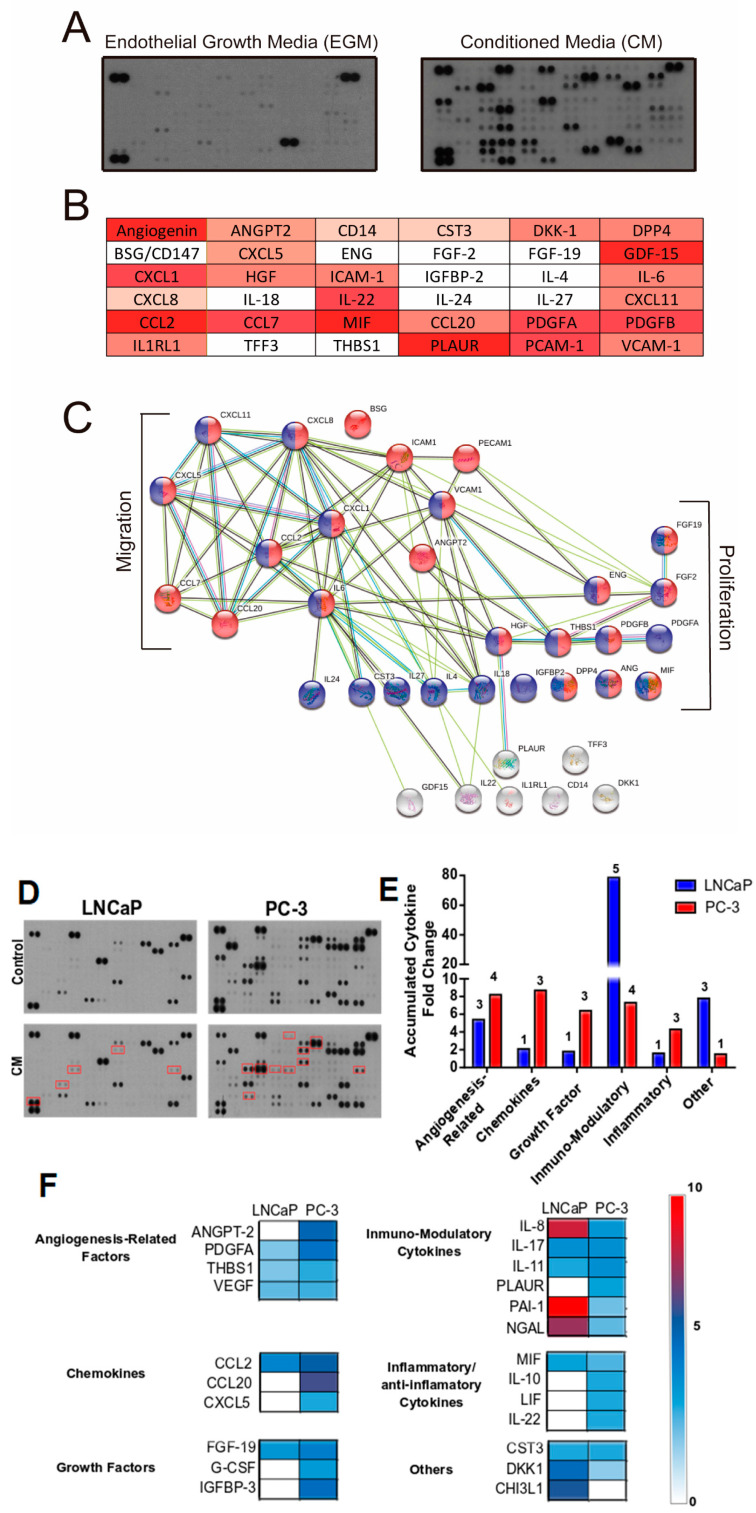
Secreted factors involved in cross-talk communications between endothelial and prostate cancer cells. Cytokine expression profiles in the CM derived from endothelial cells and cell cultures of CaP cells exposed to CM from endothelial cells. (**A**) Cytokine array of CM derived from HUVEC (right panel) and endothelial growth media “EGM” (left panel). (**B**) Graphical representation of the fold-change of cytokines in the CM derived from HUVEC. (**C**) A protein–protein interaction network map for the 36 secreted factors was generated using the STRING program. Proteins involved with cell migration (red) and cell proliferation (blue) are colored. (**D**) Cytokine array of the cell supernatants of PC-3 and LNCaP cells (Top panel) and PC-3 and LNCaP cells exposed to endothelial cell CM (bottom). The red squares indicate the factors that increase with the conditioned medium. (**E**) Graphical representation of the total accumulated fold-change of cytokines in the cell supernatant of PC-3 cells compared to LNCaP cells. (**F**) Representation of secreted proteins that are increased by incubation with endothelial cell CM. The proteins are divided according to their biological function. White squares indicate factors not increased, and dark red squares indicate the maximal induction fold (50×). The density of the images was analyzed by ImageJ software and cytokines and growth factors that demonstrated increases of 1.5-fold, or more, relative to the control were scored as positive.

To gain insight into the potential mechanisms of paracrine communication between HUVEC and PCa cells, we performed a proteomic analysis of soluble proteins in HUVEC-CM and in the supernatant of PCa cells previously exposed to HUVEC-CM (Figure 5). Our results show that 36 out of 105 intercellular communicating factors analyzed were significantly increased in the HUVEC-CM (Figure 5A,B). An analysis of the protein–protein interaction revealed that 29 out of the 36 secreted factors demonstrated functional interactive partners (Figure 5C). Consistently, these factors were correlated with cancer-associated biological process, such as cell proliferation (HGF: Hepatocyte growth factor and FGF2: Fibroblast growth factor 2), cell migration (CCL20: Chemokine (C-C motif ligand 20, CXCL5: C-X-C motif chemokine 5, IL-6: Interleukin 6) and cell communication (ICAM-1: Intercellular adhesion molecule 1) (Figure. 5B). In addition, we analyzed the profile of factors secreted by LNCaP and PC-3 PCa cells that were exposed to HUVEC-CM (Figure 5D). We observed that expression of ANGPT-2 (Angiopoietin-2), PDGFA (Platelet-derived growth factor subunit A), CXCL5 (C-X-C motif chemokine 5), G-CSF (Granulocyte-colony stimulating factor) and CCL20 were increased in PC-3 compared to LNCaP cells (Figure 5E,F). The differential expression could explain, at least partially, the variation in effect of HUVEC-CM on migration and invasion between the two PCa cell lines.

### 3.6. HUVEC-CM Induces Differential Changes in Gene Expression That Determine Aggressiveness in PCa Cells

**Figure 6:**
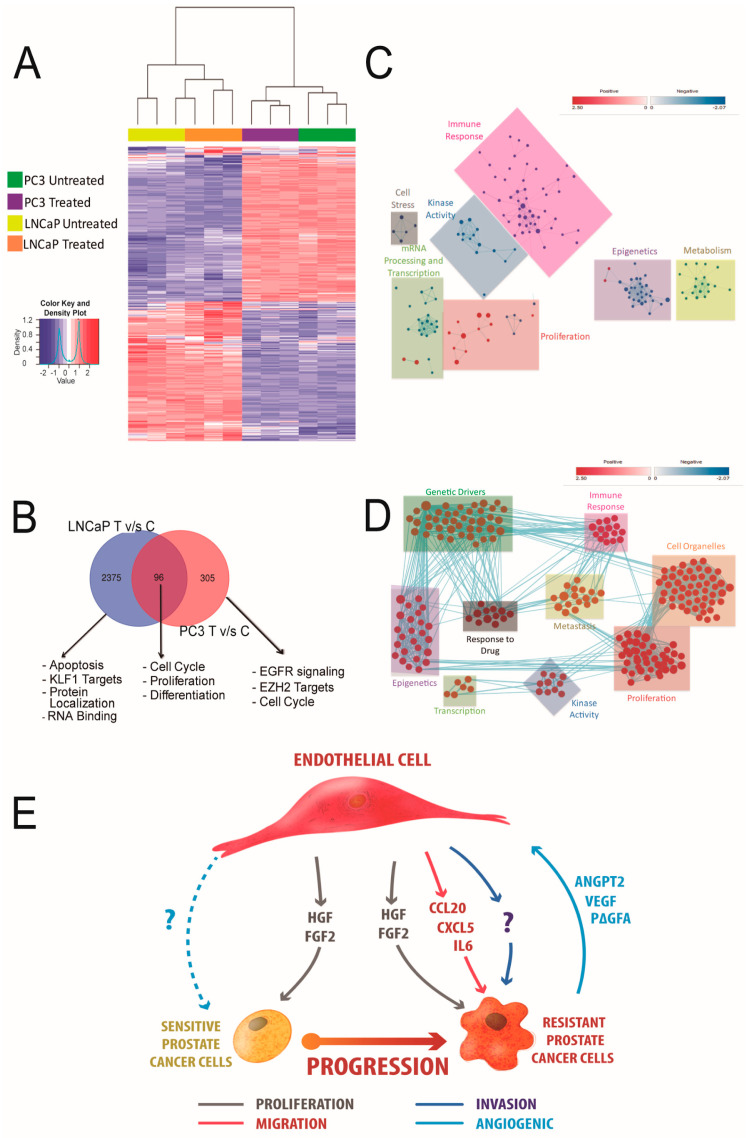
HUVEC-CM induced differential changes in gene expression in prostate cancer cell lines. RNA-seq expression analysis of PC3 and LNCaP cells exposed for 48 h to HUVEC-CM. (**A**) Heat map of differentially expressed genes produced by PC3 cells and LNCaP cells incubated with HUVEC-CM compared to their untreated controls, based on their Euclidean hierarchical clustering. (**B**) Venn diagram of overlapping genes from the DEG lists produced by comparison of LNCaP CM-treated vs. LNCaP-untreated control cells, and PC3 CM-treated vs. PC3-untreated control cells. (**C**,**D**) Network visualization of the GSEA gene clusters of LNCaP (**C**) and PC3 (**D**) differentially expressed genes. Node size indicates the level of enrichment for a particular gene set, and color indicates the degree of enrichment (red is up/treated and blue is down/untreated). (**E**) Proposed model of the factors that could be involved in communication between endothelial cells and PCa cells.

We performed transcriptome analyses of LNCaP and PC-3 cell lines treated with HUVEC-CM. As expected, the treated PC3 cells clustered with the treated LNCaP cells, and the untreated PC-3 and LNCaP cells clustered together (Figure 6A). However, differential gene expression (DGE) analysis revealed distinct gene expression patterns in HUVEC-CM-treated PCa cells compared to the untreated control cells (Figure 6B). A gene set enrichment analysis (GSEA) of the HUVEC-CM-treated LNCaP cell line showed the upregulation of pathways related only to proliferation. In contrast, HUVEC-CM-treated PC-3 cells were significantly enriched for gene sets related to metastasis, epigenetics, proliferation, genetics drivers and kinase activity pathways (Figure 6C,D). Together, these results are consistent with our in vitro and in vivo data that indicated HUVEC-CM promotes a hyperproliferative phenotype in LNCaP cells and a hyperproliferative as well invasive and metastatic phenotype in PC-3 cells (Figure 6E). Remarkably, the effect of HUVEC-CM on PC-3 cell proliferation and migration was maintained for at least 10 passages after removing HUVEC-CM stimulation, which was not observed in LNCaP cells (Supplementary Figure S4).

## 4. Discussion

Due to the failure of the anti-angiogenic therapy’s ability to increase the long-term survival of patients with PCa [8], the hypothesis that the cellular and molecular mechanism(s) by which the endothelium regulates tumor growth is broader and more complex seems reasonable. A paracrine mechanism that is independent of the proliferative state of endothelial cells as well as blood flow [10] could explain, at least partially, why this signaling activity cannot be affected by conventional anti-angiogenic therapies that target mostly endothelial cells that are in a proliferative state. Interestingly, this hypothesis reveals the need for a better understanding of the contributions of endothelial cells to PCa biology. Our results show that HUVEC-CM increased PCa cell proliferation, migration and invasion using in vitro assays and promoted tumor growth, migration and metastasis using in vivo assays. Interestingly, these effects were selective for more aggressive PCa cell lines, suggesting that more aggressive PCa cells could adapt to respond to a broader spectrum of signals derived from tumor endothelial cells. This endothelium/PCa cell interaction via the secretome of ECs could play a significant role in more advanced stages of this disease. It is interesting to note that at least part of these effects was reproduced when using HMEC-1-CM, which suggest a moderate degree of universality of the paracrine effect of endothelial cells on PCa cells. Future studies are required to compare EC secretomes in order define whether HUVEC/HMEC-1-CM and HCMEC-D3 secrete a differential pattern of chemokines and growth factors.

Even though our study does not contemplate the use of endothelial cells isolated from human prostate cancer [28,32,33], HUVECs have been widely used to model molecular interactions between endothelial and cancer cells in various tumor models [10,11,16]. In addition, previous studies from our laboratory [24] have shown that HUVEC cells can recapitulate certain functional characteristics similar to endothelial cells isolated from human prostate tumor tissue. An example of this is their androgen responsiveness, a capability that only a few vascular beds possess in the human body, and is based on their ability to express the androgen receptor [24]. Taking these aspects into account, we believe that HUVEC cells represent a valid model for the study of the potential interaction between endothelial cells and prostate tumor cells. As a result of the moderate degree of universality of the effects using other endothelial cell lines, we highlight the need to further explore the use of alternative endothelial cell models that complement these results.

Previous studies in breast cancer and leukemia models indicated that the effect of the endothelium on malignant cells to promote tumor growth and metastatic potential, as well as the expansion of the primitive leukemia-initiating cell pool, respectively, requires a direct contact between both cell types [19,20]. Studies in hepatocellular and colorectal cancers showed that CM from endothelial cells represent a sufficient stimulus to produce similar effects [16,34]. We demonstrated that most of the effect of endothelial cells on PCa cells were mediated through a paracrine mechanism(s) with little to no additional contribution by the physical interaction of both cell types.

Our in vitro studies showed that, unlike benign RWPE-1 cell line, all PCa cell lines analyzed responded to signals from endothelial cells by increasing their proliferation. Together, these observations highlight the difference between tumor cells and non-malignant cells in terms of their ability to respond to endothelial cell-derived signals. However, HUVEC-CM caused a differential effect in terms of its ability to affect the migration and invasion capacities of PCa cells. Remarkably, our results demonstrate that only the most aggressive PCa cells (LNCaP-C4-2 and PC-3) showed a significant increase in their ability to migrate and invade in response to HUVEC-CM. This effect was not observed in the less aggressive androgen-sensitive LNCaP cell line or the non-tumorigenic RWPE-1 cell line. This differential effect was also observed in the zebrafish in vivo model. Previous studies using a CR-PCa cell line showed that there was an increase in the incidence of metastasis to different organs in the zebrafish when PCa cells were co-injected with HUVEC [21]. Our results using both the zebrafish and immunocompromised mouse models as hosts for tumor transplantation replicate these results and show that a single treatment of PCa cells with HUVEC-CM before injection into the animal models was sufficient to promote tumor growth and migration to levels similar as when the PCa cells were co-injected with HUVEC cells. Overall, this evidence supports the idea that the effect of endothelial cells is broader in more aggressive PCa cells. The enhanced response could reflect either a greater sensitivity of these cells to signal molecules in the conditioned medium or an increased/amplified intracellular capacity to respond to comparable levels of signals, resulting in increased tumor growth and progression. Future studies are necessary to define whether PCa cells with increasing aggressiveness potential show a differential expression of receptors/signal transduction systems for activating or repressing factors relevant to each of these biological processes. 

Bi-directional cross-talk between endothelial and malignant cells promotes the reciprocal growth factor exchange that can influence a pro-angiogenic response [35]. We observed that tumors produced by PC-3 cells treated with HUVEC-CM or PC-3 cells co-injected with HUVEC developed an increase in the number of blood vessels that was concomitant with the level of increase in VEGF-A expression. Notably, the increase in VEGF-A was not as pronounced as expected, suggesting that tumor angiogenesis could be modulated by more than one pro-angiogenic factor. More specific proteomic analyses could help to define the profile of pro-angiogenic factors stimulated in PCa cells by endothelial cells.

To gain insight into the molecular mechanisms that mediate the biological effects of endothelial cells on PCa cells, we identified factors expressed and secreted into the CM by HUVEC cells and found that CCL2, IL-6, CXCL1, FGF2 and HGF were central nodes within the entire network of endothelial cell-secreted factors. Conversely, we analyzed the factors secreted by LNCaP and PC-3 PCa cells in the absence or presence of endothelial cell CM and found substantial and consistent differences between the PCa cell lines. LNCaP cells stimulated by HUVEC-CM mainly secrete proteins related to immune modulation (IL-8, IL17A, IL-11, Lipocalin-2 (NGAL) and SERPIN1 (PAI-1)). Among these, NGAL [36] has been correlated with promoting cell proliferation, and IL-8 correlated with reduction in the dependence of PCa cells on androgens for growth [37]. In PC-3 cells, HUVEC-CM stimulated the secretion of factors related to inflammation (IL-10 and Il-22), chemokines (MIP3A and CXCL5) and factors associated with angiogenesis (THBS1 and ANG-2) that are related to the promotion of cell survival, proliferation, enhancing the growth of xenografted tumor cells, tumor vascularization and invasion in vivo [38,39]. These differences between the two cell lines could partially explain why endothelial-cell CM-stimulated PC3 xenograft tumors have higher microvascular density and metastatic capability.

To unravel the differences between the response of LNCaP and PC-3 cells exposed to CM, we carried out RNA-seq analyses. Genes that were differentially expressed between LNCaP and PC-3 cells in presence of HUVEC-CM could explain the biological behavior of the two cell lines. In LNCaP cells, the profile of DEGs was enriched for cellular processes related to apoptosis, proliferation and RNA binding, whereas in PC-3 cells, the DEGs were enriched for cellular processes related to metastasis and cell cycle. It is noteworthy that the invasive phenotype induced by HUVEC-CM in PC3 cells was maintained even when the cells were no longer exposed to signaling induced by the HUVEC-CM. Consistent with this observation, we found a strong enrichment of gene sets associated with epigenetic modulation in PC3 cells exposed to endothelial cell HUVEC-CM. Future studies should interrogate the hypothesis that signaling pathways induced in PC3 cells by HUVEC-CM lead to the epigenetic reprogramming of pathways associated with cell migration and invasion, and their potential underlying mechanisms. Our analyses indicated that endothelial cells increased PCa cell proliferation, migration and invasion in vitro, and tumor growth and metastasis in vivo. Interestingly, the paracrine role of endothelial cells on PCa cell biology was higher and broader in more aggressive PCa phenotypes, suggesting that these phenotypes could adapt by increasing sensitivity to extracellular signals in the tumor microenvironment. Accordingly, tissue-specific metastasis reinforces the fact that endothelial–tumor cell interactions can differentially affect the tumor microenvironment. Additionally, our data indicated that paracrine secretion, and not direct cell–cell contact, was a sufficient stimulus to mediate the biological effects of endothelial cells on PCa cells. It is important to highlight that, because of the way we obtained the HUVEC-CM [11,25,26], we cannot exclude the possibility of molecules other than growth factors, such as exosomes and miRNAs, could have contributed to the biological effect of HUVEC on PCa cells. Further studies on cytokines/growth factor(s) released by endothelial cells, as well as the molecular pathways regulated in PCa cells as a result of this interaction, could culminate in the identification of these molecular interactions, which could provide new biomarkers or therapeutic targets to counteract PCa, especially advanced PCa.

## 5. Conclusions

Our results allow us to conclude that paracrine stimulation by HUVEC cells on PCa cells increased their in vitro proliferative activity and in vivo tumor growth and selectively promoted migration in vitro and in vivo and metastatic potential in vivo in more aggressive PCa cell lines.

## Data Availability

The data presented in this study are available in this article and supplementary material.

## Supplementary Materials

The following supporting information can be downloaded at: https://www.mdpi.com/article/10.3390/cancers14194750/s1, Supplementary Material and Methods S1; Figure S1. HUVEC-CM increases the proliferation of PCa cell lines; Figure S2. HUVEC-CM increases migration in aggressive PCa cell lines. Figure S3. HMEC-1-CM increases the migration of cancer cell lines. Figure S4. Effect of HUVEC-CM on LNCaP and PC-3 cell proliferation and migration in 7 passages after removing HUVEC-CM stimulation. Table S1. A schematic representation of the cytokine/chemokine spot positions in duplicate on the membrane with respective internal controls.
