# Supplementary material for: The Differential Paracrine Role of the Endothelium in Prostate Cancer Cells
Source: Cancers (Basel). 2022 Sep 29;14(19):4750. doi: 10.3390/cancers14194750 (PMC9563990; doi:10.3390/cancers14194750)
Supplement: Supplementary file 1 [file cancers-14-04750-s001.zip › cancers-1875354-supplementary.pdf]

Supplementary Information

Differential Paracrine Role of Human Umbilical Cord Endothelial Cells on Prostate Cancer Cells

Verónica Torres-Estay[4], Michalis Mastri[10], Spencer Rosario[10], Patricia Fuenzalida[1], Carolina E. Echeverría[5,6], Emilia Flores[5], Anica Watts[10], Javier Cerda-Infante[3], Viviana P. Montecinos[3], Paula C. Sotomayor[2], Julio Amigo[1], Carlos A. Escudero[7,8], Francisco Nualart[9], John M. L. Ebos[11,12], Dominic J. Smiraglia[11], Yue Wu[10], and Alejandro S. Godoy[5,10,*]

Departments of Physiology[1], Urology[2], and Hematology Oncology[3], Pontificia Universidad Católica de Chile, Santiago, Chile; Department of Chemical and Biological Sciences[4], Universidad Bernardo O'Higgins, Santiago, Chile; Centro de Biología Celular y Biomedicina (CEBICEM)[5], Facultad de Medicina y Ciencia, Universidad San Sebastián, Santiago, Chile; Centro de Investigación e Innovación Biomédica[6], Universidad de los Andes, Santiago, Chile; Departamento de Ciencias Básicas[7], Universidad del Bio-Bio, Chillán, Chile; Group of Research and Innovation in Vascular Health (GRIVAS Health)[8], Chillan, Chile; Departamento de Biología Celular[9], Facultad de Ciencias Biológicas, Universidad de Concepción, Concepción, Chile; Departments of Urology[10], Cancer Genetics and Genomics[11], and Medicine[12], Roswell Park Comprehensive Cancer Center, Buffalo NY, 14263, USA.

Supplementary Material and Methods S1

Cell Cultures Conditions: All HUVEC cells were cultured in SFM-endothelial medium (GIBCO) supplemented with 10% fetal bovine serum (FBS) and 10 µg/ml of endothelial growth factor (Merck). Cell culture media for each cell line were used as follows: RPMI-1640 media (GIBCO) for LNCaP-C4-2 and PC-3 cell lines, RPMI-1640 media supplemented with 1 nM of dihydrotestosterone (DHT) for LNCaP cell line and Keratinocyte Serum Free Medium (pK-SFM, GIBCO) for RWPE-1 cell line. All media were supplemented with heat-inactivated fetal bovine serum (10%), 100 µg/ml streptomycin and 100 U/ml penicillin. Cell cultures were maintained at 37°C with 5% CO_2 in a humidified incubator.

Animals models: Immunocompromised male NOD.*Cg-Prkdc*^{scid} *Il2rg*^{tm1Wjl}/SzJ (NSG) mice were acquired from The Jackson Laboratory, USA, and housed under specific pathogen-free conditions and 12 h day/night cycles at the animal facility of PUC. The Zebrafish (Danio rerio) model was acquired from Zebrafish International Resource Center (ZIRC, USA) and kept at 28°C with a 14/10 h day/night cycle at the animal facility from PUC. Embryos raised beyond 24 h post-fertilization (hpf) were treated with phenylthiourea (PTU; 0.003%, w/v; Sigma) to make them translucent. All animal protocols were conducted under the approval of the ethical committee of PUC.

Cell proliferation assays: PCa cell lines were seeded at a density of $1x10^3$ cells/well in 96 well plates and incubated overnight. After that, culture media was replaced with HUVEC-CM in different proportions mixed with culture media supplemented with 5% FBS for 96 h. The media was replaced every 48 h. The number of viable cells was determined using CellTiter 96 aqueous non-Radioactive Cell Proliferation kit (Promega) assay according to the manufacturer's instructions. The optical density was measured at 570 nm using an ELISA reader (EL800, BioTek Instruments, Winooski, VT). Cell survival rate was expressed as A/B

× 100, where A was the absorbance value from the cell treated with CM and B was the absorbance value from control (untreated) cells. For Ki-67 immunofluorescence staining, PCa cell lines were cultured on coverslips to the appropriate density. After that, culture media was replaced with HUVEC-CM in a proportion of 50/50 with regular culture media for 96 h replacing this media every 48 h. Cells were fixed in 4% paraformaldehyde and then permeabilized with 0.5% Triton X-100 for 10 min. Fixed and permeabilized cells were blocked with 5% w/v BSA for 30 min and incubated with rabbit anti-Ki-67 (1:500, Abcam) antibodies overnight at 4°C. Immunostaining was then visualized by incubating the cells in a secondary antibody Alexa Fluor 488-conjugated goat anti-rabbit IgG (Invitrogen) for 1 h at room temperature. Nuclei were counterstained using 4,6-diamidino-2-phenylindole (DAPI) (1:20000, Sigma-Aldrich). Lastly, cells were photographed using a fluorescence microscope (DM IL LED, Leica).

Transwell migration and invasion assays: prostate endothelial cell lines were put into starvation overnight and then trypsinized and suspended in RPMI medium or CM without FBS at 5×10^4 cells/ml for the migration assay and at 2×10^5 cells/ml for the invasion assay. Cell suspension (100 μl) was added to the upper well, and 150 μl of RPMI media containing 10% FBS was added to the lower well. Cells in the wells were incubated for 24 h for migration assay and 48 h for invasion assay. Lastly, migrated/invaded cells were dissociated from the membrane and subsequently detected with CyQuant® GR Dye. Fluorescence was quantified using a fluorescence plate reader at 480 nm/520 nm (Synergy™ 2 Multi-Detection Microplate Reader, BioTek Instruments, Inc).

Zebrafish xenograft model: LNCaP and PC-3 cells were fluorescently labeled using CellTracker™ Red CMTPX (Invitrogen, Life Technologies, Carlsbad, CA, USA) according to the manufacturer's instructions. Labeled cells were washed in PBS twice and re-

suspended in PBS/EDTA at a concentration of 1×10^6 cells/ml. For the microinjection of labeled cells into the embryos, zebrafish embryos of 2 days post-fertilization (dpf) were dechorionated and anesthetized with tricaine (Sigma, USA). Embryos were injected into the middle of the embryonic yolk sac region with approximately 250 PCa cells and then incubated at 28°C. Embryos were imaged individually at 3 days post-implantation under an inverted wide-field fluorescence microscope (DM IL LED, LEICA). Tricaine (0.04 mg/ml) was added to their water, and then the embryos were mounted on a slide with 0.5% agarose to prevent their movement during the live imaging process. Cell fluorescence (red pixels) was measured in the tail of the fishes and quantified using ImageJ software.

Cell line-derived xenograft model: NSG mice were subcutaneously injected at the upper left flank region with 0.1 ml of PC-3 cell suspension (in phosphate-buffered saline containing 50% Matrigel) containing $1x10^6$ PC-3 cells for control conditions, $1x10^6$ PC-3 cells preincubated with HUVEC-CM for 48 h, or a mixture of $1x10^6$ PC-3 cells and $2x10^5$ HUVECs cells (5:1). Tumor growth was evaluated by measuring the length and width of tumor mass at the inoculation site with a caliper. The tumor volumes were determined using the following formula $V= w^2l/2$, where “w” is the width, and “l” is the large of tumors. After 7 weeks, the tumor-bearing mice were sacrificed. The tumors, kidneys, lungs, and liver were removed and fixed in 10% phosphate-buffered formalin and were embedded in paraffin for pathological analysis.

Immunohistochemistry: Briefly, after rehydration and antigen retrieval, tissue slides were incubated with primary antibodies against Ki-67 (1:100, Abcam), CD31 (1:100; Santa Cruz Biotechnology), cleaved caspase-3 (1:100, Cell signaling) or VEGF (1:300; Santa Cruz Biotechnology), followed by an HRP-conjugated anti-rabbit or anti-mouse secondary antibody (1:200, Dako). Immunostaining in the absence of primary antibodies provided

negative controls. Immunostaining was revealed using an Impact NovaRED substrate kit (Vector Lab) according to the manufacturer's protocol.

Expression analysis: PCa cell RNA was isolated using a RNeasy kit (Qiagen). 500 ng of total RNA was converted to cDNA, followed by *in vitro* transcription to generate biotin-labeled cRNA using the Ambion Illumina Total Prep RNA Amplification (Ambion, Inc) as described in the manufacturer's instructions. Differential gene expression analysis of RNA sequencing was conducted using log-transformed fragments per kilobase per million mapped fragments (FPKM+1) with the Bioconductor Limma package in the statistical analysis software "R". A Bayesian empirical model was then constructed based upon the transformed FPKM data and compared to generate Differentially Expressed Gene (DEG) lists. Genes with an adjusted *p*-values of less than 0.05 and greater than a 1.5-fold change/less than a -1.5 fold change in expression were considered differentially expressed. DEGs were then used to construct a pre-ranked list for Gene Set Enrichment Analysis (GSEA). Ranks were assigned by multiplying the -log (adjusted p value) and the fold change, together. GSEA pre-ranked was then performed on these lists of enriched pathways. The results of the GSEA analysis were then used to generate networks in Cytoscape, with the implementation of EnrichmentMap and AutoAnnotate plug-ins.

Supplementary Figure and Table Legends

Figure S1. HUVEC-CM increases the proliferation of PCa cell lines. A) Immunostaining analysis of the proliferation marker Ki-67 in prostate cell lines exposed to CM (50/50). DAPI was used to counterstain nuclei. B) Quantitation of the Ki-67 immnunostaining data. Ki-67 positive cells were estimated as a percentage of total number of nuclei (determined by DAPI staining) (n=3; **p* ≤ 0.05, **p ≤ 0.01 t-test)

Figure S2. HUVEC-CM increases migration in aggressive PCa cell lines. A) Representative phase microscopic images of wound healing assay of control RWPE-1, LNCaP, PC-3 and LNCaP-C4-2 cells at the end of the observation period (16 hrs for RWPE-1 and LNCaP; and 8 hr for PC-3 and LNCaP-C4-2 cells) in basal condition (Control) and using HUVEC-CM. Black lines represent the area at time 0 hrs. B) Quantification of cell migration using the monolayer wound healing assay. The areas covered by migrated cells were quantified by Image-J (**P* ≤ 0.05; n=3 t-test).

Figure S3. HMEC-1-CM increases migration of cancer cell lines. A) Analysis of the effect of CM isolated from HMEC-1 and HCMEC-D3 cell line culture after mixture with standard PCa cell media supplemented with 5% FBS, on cell proliferation of LNCaP and PC3 cells using the MTT assay. Control conditions for all experimental approaches was fresh endothelial culture medium (without FBS or growth factors) added to the standard RMPI medium in a 50/50 proportion (**p* ≤ 0.05; n=3) B) Quantification of cell migration using the monolayer wound healing assay. The areas covered by migrated cells were quantified by Image-J (**P* ≤ 0.05; n=3 t-test).

Figure S4. Effect of HUVEC-CM on LNCaP and PC-3 cell proliferation and migration 7 passages after removing HUVEC-CM stimulation. A) MTT Analysis of the effect of HUVEC-CM on cell proliferation of LNCaP and PC3 cells 7 passages after removing the initial stimulus. B) Representative phase microscopic images of wound healing assay of LNCaP and PC-3 in basal condition (Control) and using HUVEC-CM 7 passages after removing the initial stimulus. Black lines represent the area at time 0 hrs. Plots on the right side of the panel represent the quantification of cell migration using the monolayer wound healing assay. The areas covered by migrated cells were quantified by Image-J (*$P \leq 0.05$; n=3 t-test).

Supplementary Table S1: A schematic representation of the cytokine/chemokine spot positions in duplicate on the membrane with respective internal controls. (Proteome Profiler Human XL Cytokine Array Kit (R&D systems))

Supplementary Figures

Supplementary Figure S1

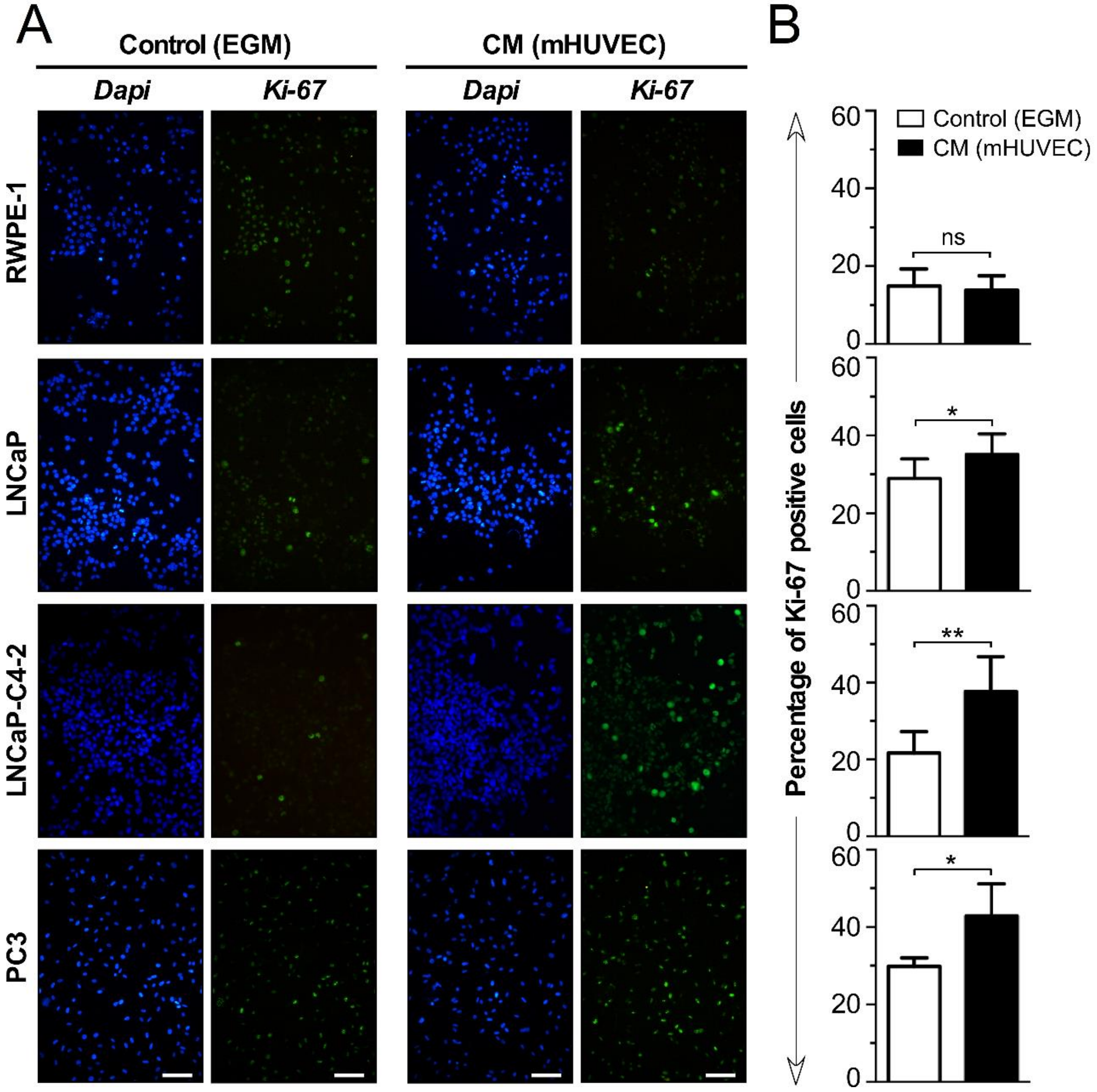

Supplementary Figure S2

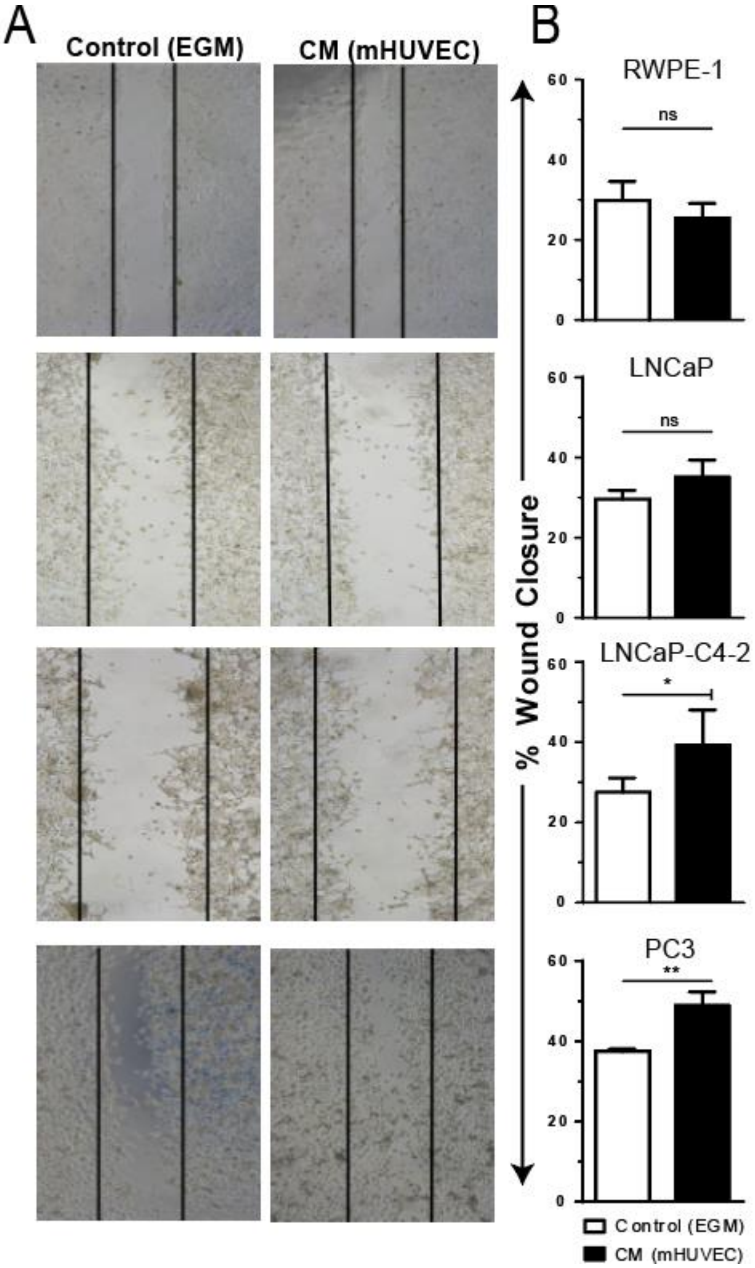

Supplementary Figure S3

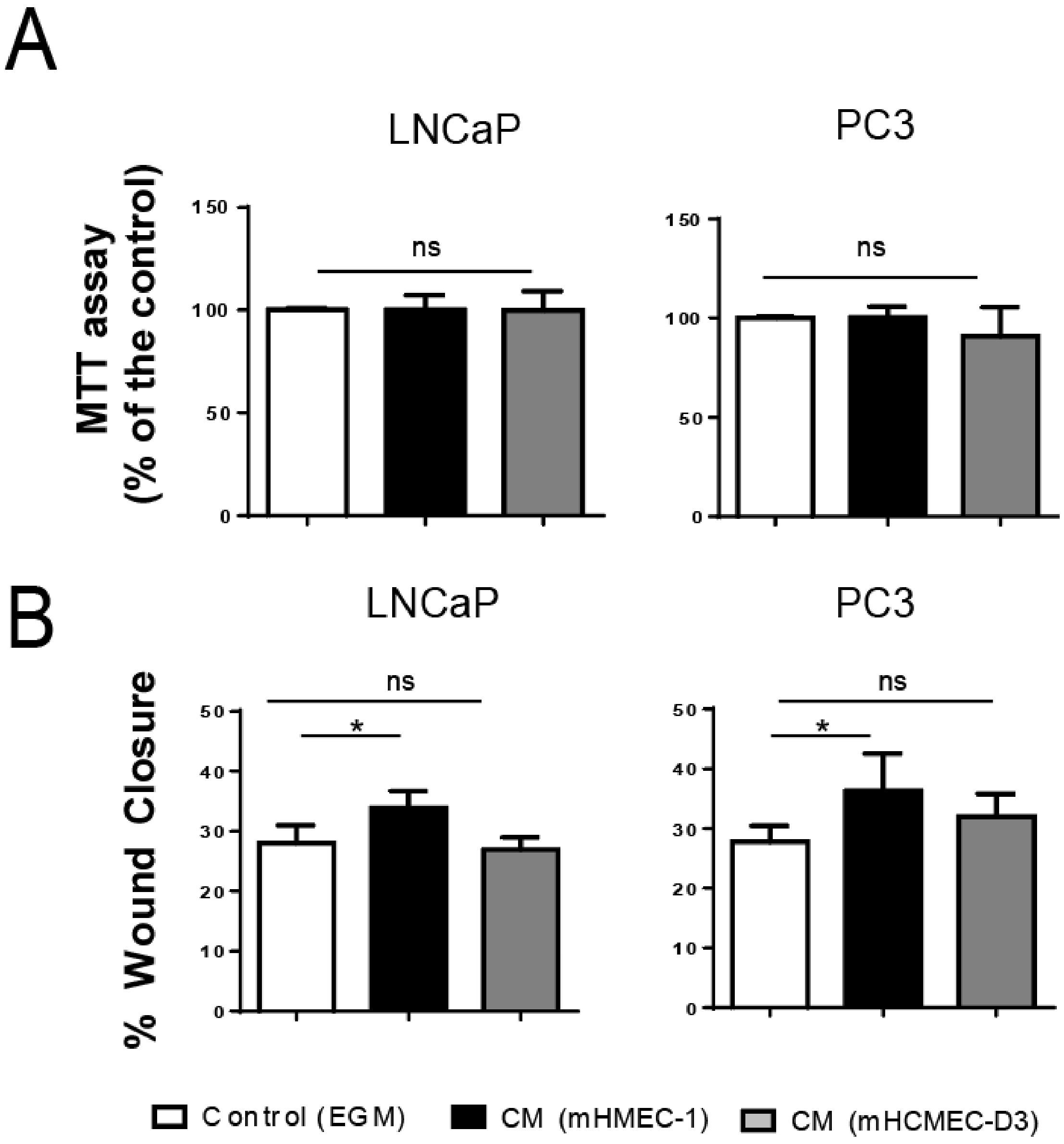
A
LNCaP
PC3
MTT assay
(% of the control)
150
100
50
0
ns
B
LNCaP
PC3
% Wound Closure
50
40
30
20
10
0
ns
*
Control (EGM)
CM (mHMEC-1)
CM (mHCMEC-D3)

Supplementary Figure S4

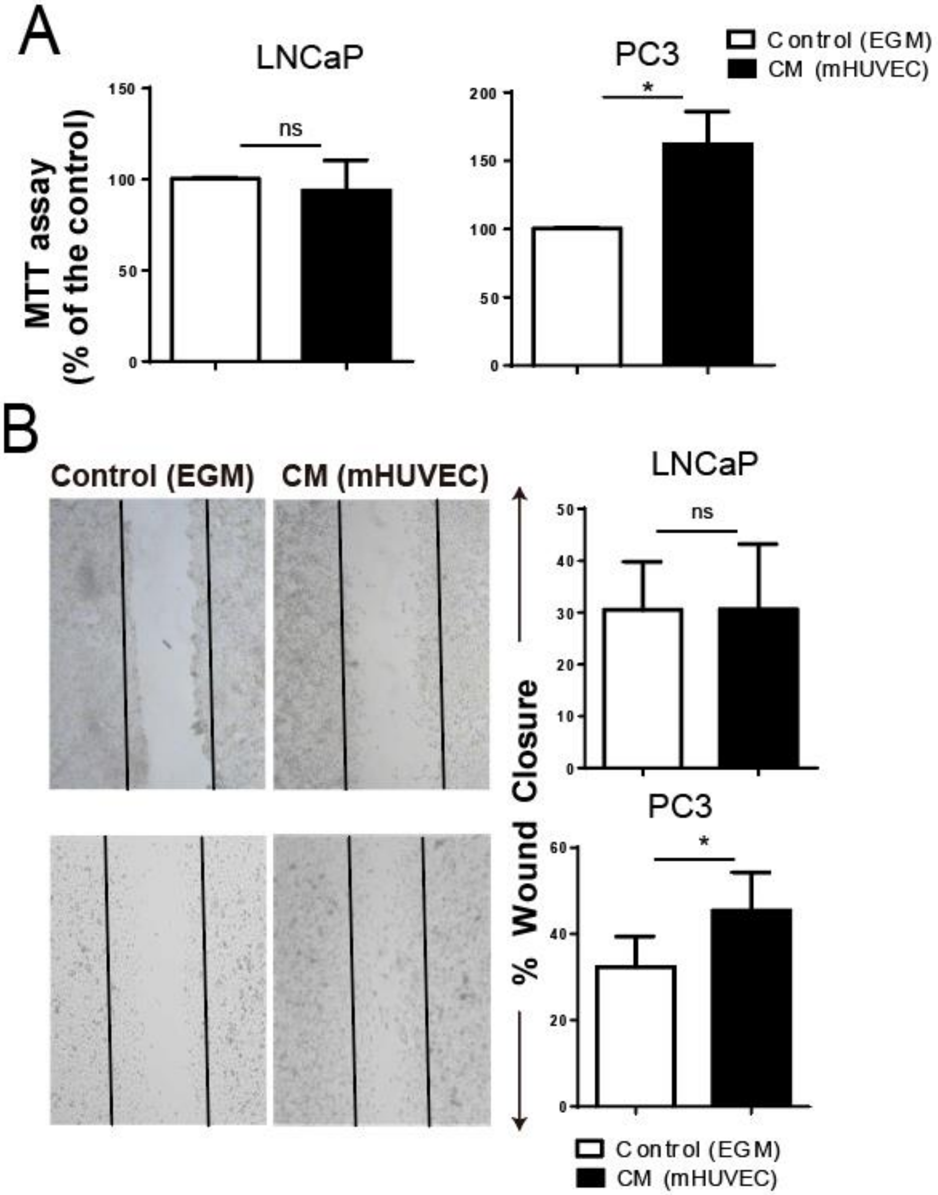

Supplementary Table

Supplementary Table S1.

	1 2	3 4	5 6	7 8	9 10	11 12	13 14	15 16	17 18	19 20	21 22	23 24
A	Reference	Adiponectin	ApoA1	Angiogenin	ANGPT-1	ANGPT-2	BAFF	BDNF	C5/C5a	CD14	CD30	Reference
B		CD40 L	CHI3L1	CFD	CRP	Cripto-1	CST3	Dkk-1	DPPIV	EGF	BSG/CD147	
C		CXCL5	ENG/CD105	Fas Ligand	FGF-2	KGF/FGF-7	FGF-19	Flt-3 Ligand	G-CSF	GDF-15	GM-CSF	
D	CXCL1	GH	HGF	ICAM-1	IFN-gamma	IGFBP-2	IGFBP-3	IL-1 alpha	IL-1 beta	IL-1ra	IL-2	IL-3
E	IL-4	IL-5	IL-6	CXCL8	IL-10	IL-11	IL-12 p70	IL-13	IL-15	IL-16	IL-17A	IL-18 Bpa
F	IL-19	IL-22	IL-23	IL-24	IL-27	IL-31	IL-32	IL-33	IL-34	CXCL10	CXCL11	PSA
G	Leptin	LIF	NGAL	CCL2	CCL7	M-CSF	MIF	CXCL9	CCL3	CCL20	CCL19	MMP-9
H	MPO	OPN	PDGF-AA	PDGF-AB/BB	TSF-14	CXCL4	RAGE	CCL5	RBP4	Relaxin-2	Resistin	CXCL12
I	PAI-1	SHBG	IL1RL1	CCL17	TFF3	TfR	TGF-alpha	THBS1	TNF-alpha	uPAR/PLAUR	VEGF	
J	Reference		Vitamin D	PECAM-1	TIM-1	VCAM-1						Negative
